# Copper homeostasis and cuproptosis in atherosclerosis: metabolism, mechanisms and potential therapeutic strategies

**DOI:** 10.1038/s41420-023-01796-1

**Published:** 2024-01-13

**Authors:** Shengjie Yang, Yujuan Li, Lijun Zhou, Xinyue Wang, Longtao Liu, Min Wu

**Affiliations:** 1 Guang’an men Hospital, China Academy of Chinese Medical Sciences, Beijing, 100053 China; 2 Xiyuan Hospital, China Academy of Chinese Medical Sciences, Beijing, 100091 China

**Keywords:** Cell death, Cardiovascular diseases

## Abstract

Copper is an essential micronutrient that plays a pivotal role in numerous physiological processes in virtually all cell types. Nevertheless, the dysregulation of copper homeostasis, whether towards excess or deficiency, can lead to pathological alterations, such as atherosclerosis. With the advent of the concept of copper-induced cell death, termed cuproptosis, researchers have increasingly focused on the potential role of copper dyshomeostasis in atherosclerosis. In this review, we provide a broad overview of cellular and systemic copper metabolism. We then summarize the evidence linking copper dyshomeostasis to atherosclerosis and elucidate the potential mechanisms underlying atherosclerosis development in terms of both copper excess and copper deficiency. Furthermore, we discuss the evidence for and mechanisms of cuproptosis, discuss its interactions with other modes of cell death, and highlight the role of cuproptosis-related mitochondrial dysfunction in atherosclerosis. Finally, we explore the therapeutic strategy of targeting this novel form of cell death, aiming to provide some insights for the management of atherosclerosis.

## Facts

1. Copper is an essential trace element required in various physiological processes in the human body.
2. Dysregulation of copper homeostasis, whether towards excess or deficiency, has been implicated in various health problems, including atherosclerosis.
3. Dysregulation of copper homeostasis and copper-induced cell death (cuproptosis) are acknowledged as potential contributors to the pathogenesis of atherosclerosis.

## Open questions

1. What is the safe window for copper levels to avoid the development of atherosclerosis? How can the suitable copper concentration be determined for the treatment of atherosclerosis?
2. What are the potential candidate biomarkers that can reliably and sensitively indicate the occurrence of cuproptosis in the context of atherosclerosis?
3. What are the potential undiscovered roles of copper in mitochondrial function, and is there interplay between copper and mitochondrial dynamics or mitophagy?

## Introduction

Atherosclerosis is a chronic, progressive disease characterized by the accumulation of lipids, inflammatory cells, and fibrous elements in the arterial wall, leading to the formation of atherosclerotic plaques [1, 2]. These plaques can lead to serious clinical consequences, such as myocardial infarction (MI) and stroke, which are major causes of morbidity and mortality worldwide [3]. The pathogenesis of atherosclerosis involves multiple genetic, environmental, and metabolic factors [4].

Copper is an essential trace element involved in mitochondrial respiration, antioxidant defense, and neurotransmitter synthesis [5, 6]. Copper homeostasis is tightly regulated. Copper excess or deficiency can lead to pathological alterations, and thus negatively affect human health [7]. Copper dyshomeostasis may contribute to the pathogenesis of atherosclerosis by increasing oxidative stress, inflammation, endothelial dysfunction, and lipid metabolism levels [8–10]. Moreover, a novel type of cell death, which is copper-dependent, has recently been described. This copper-induced cell death, termed cuproptosis, may contribute to atherosclerosis development by causing cell death and impairing mitochondrial function [11, 12].

In this review, we explore the role of copper homeostasis and cuproptosis in atherosclerosis. We also discuss potential therapeutic strategies against cuproptosis and provide directions for future research on cuproptosis and atherosclerosis.

## Copper metabolism

### Systemic copper homeostasis

**Fig. 1:**
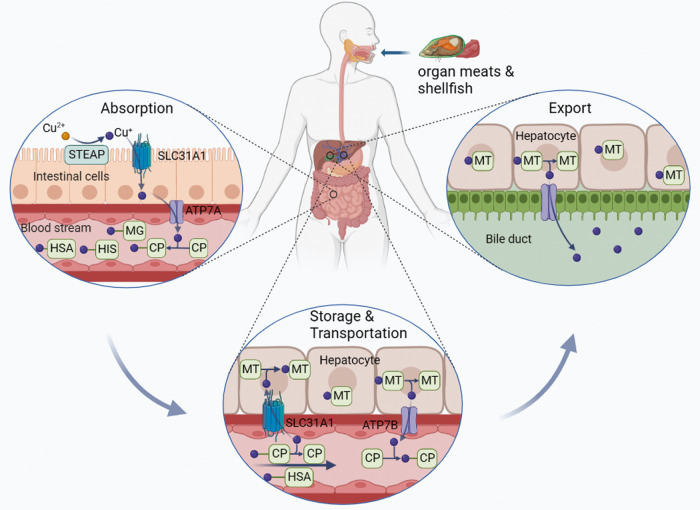
Schematic diagram of systemic copper metabolism process. Copper absorption occurs primarily in the small intestine, a process mediated by SLC31A1. Copper is then transported and exported to the bloodstream through the action of ATP7A, combined with soluble chaperones, and transported through the portal system to the liver, where it is stored and further transported. Excess copper is excreted by the liver into the bile. Figure created with BioRender. SLC31A1 solute carrier family 31 member 1, ATP7A and ATP7B ATPase copper transporters 7 A and 7B, STEAP six-transmembrane epithelial antigen of the prostate, HSA human serum albumin, CP ceruloplasmin, MT metallothionein, HIS histidine, MG macroglobulin.

Copper is an essential trace element required in various physiological processes in the human body. Research shows that Cu levels vary across organs and tissues, with values ranging from 3 mg (kidneys) to 46 mg (bone) [13]. Copper serves as a cofactor for numerous enzymes involved in energy metabolism, neurotransmitter synthesis, and antioxidant defense [5, 6, 14]. Excessive or deficient copper levels can, however, lead to cytotoxicity and pathological alterations [7]. Therefore, it is essential to maintain systemic copper levels within a narrow range **(**Fig. 1**)**.

#### Copper absorption

Copper is mainly obtained from dietary sources such as organ meat and shellfish [15]. Copper is primarily absorbed by enterocytes in the small intestine, and its uptake is mediated by copper transporter 1 (CTR1), also known as solute carrier family 31 member 1 (SLC31A1) [16]. CTR1 appears to play a primary role in this uptake, as indicated by research showing greatly reduced copper accumulation in the peripheral tissues of neonatal mice lacking CTR1 [17]. The six-transmembrane epithelial antigen of the prostate (STEAP) facilitates this process by reducing divalent Cu^2+^ to Cu^+^.

#### Copper storage and transportation

After uptake by the intestinal epithelial cells, copper is transported and exported into the bloodstream by the ATPase copper transporter 7 A (ATP7A) [18]. Here, copper is transported through the portal system to the liver by binding to soluble chaperones such as human serum albumin (HSA), ceruloplasmin, histidine, and macroglobulin [19, 20]. Copper uptake by the hepatocytes in the liver is also mediated by CTR1. The liver is crucial in regulating copper metabolism by storing and excreting copper. Copper storage is mediated by the copper-binding protein metallothionein (MT), a reducing molecule rich in thiol groups with a high affinity for copper ions [21]. MT plays a crucial role in copper homeostasis by storing and releasing excess copper when required. Copper transport is performed by ATPase copper transporter 7B (ATP7B) in hepatocytes, which pumps copper ions from the liver back into the bloodstream. Here, copper ions bind to their soluble partners and are transported to specific tissues and organs [22].

#### Copper elimination

Copper is primarily eliminated via biliary excretion [7]. Excess copper is secreted by the liver into the bile and then excreted through feces [23]. The ATP7B transporter regulates the elimination of copper from the liver to bile canaliculi [24]. In cases of ATP7B dysfunction, such as Wilson’s disease, copper accumulates in the liver, leading to liver damage and subsequent health issues [25].

In summary, copper metabolism is a complex process that involves multiple mechanisms to regulate copper absorption, transport, storage, and elimination.

### Cellular copper homeostasis

**Fig. 2:**
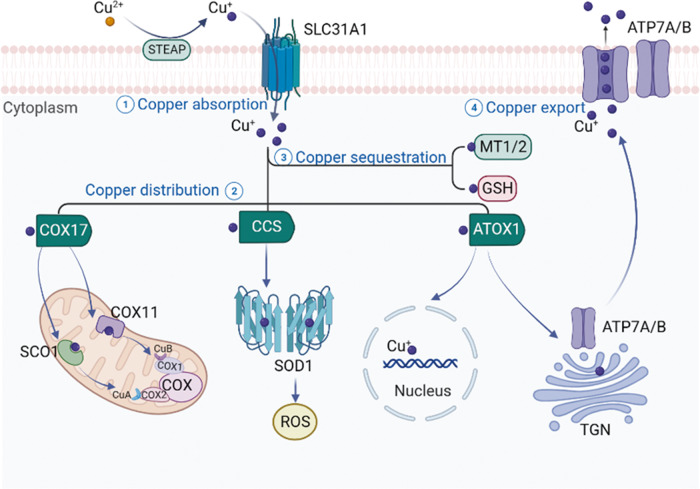
Schematic diagram of cellular copper metabolism. The maintenance of intracellular copper homeostasis is a complex and tightly regulated process. Copper ions are primarily taken up by cells via SLC31A1, whereas their export is facilitated by ATP7A/B. Once inside the cell, copper is transported to interact with cytoplasmic copper chaperones, including COX17, CCS, and ATOX1. These chaperones transport copper to specific cellular compartments, such as the mitochondria, TGN, and nucleus. Figure created with BioRender. SLC31A1 solute carrier family 31 member 1, ATP7A and ATP7B ATPase copper transporter 7A and 7B, ATOX1 antioxidant 1 copper chaperone, CCS copper chaperone for superoxide dismutase, COX17 cytochrome c oxidase copper chaperone 17, COX11 cytochrome c oxidase copper chaperone 11, COX cytochrome c oxidase, ROS reactive oxygen species, SCO1 synthesis of cytochrome c oxidase 1, SOD1 superoxide dismutase 1, TGN trans-Golgi network, GSH glutathione, MT1/2 metallothionein 1/2, and STEAP six-transmembrane epithelial antigen of the prostate.

The maintenance of intracellular copper homeostasis is a complex and tightly regulated process involving the coordinated action of copper transporters, chaperones, and cuproenzymes **(**Fig. 2**)**. Copper concentration is maintained within a narrow range through the collaborative action of these copper-dependent proteins.

#### Copper uptake

In mammalian cells, copper uptake is primarily mediated by CTR1 [6], a transmembrane protein that forms a trimeric channel to facilitate the passage of Cu ions across the plasma membrane [26]. CTR1 expression is regulated by copper levels. Low copper levels upregulate CTR1 expression to increase copper uptake, whereas high levels downregulate CTR1 expression to prevent copper cytotoxicity [27, 28]. These findings highlight the importance of CTR1 in copper homeostasis.

#### Intracellular copper distribution

After entering the cell, copper is first delivered to cytoplasmic copper chaperones, which then transport it to intracellular compartments such as the mitochondria, trans-Golgi network (TGN), and nucleus. In mammalian cells, three major copper chaperones have been identified: antioxidant 1 copper chaperone (ATOX1), copper chaperone for superoxide dismutase (CCS), and cytochrome c oxidase copper chaperone 17 (COX17) [29, 30].

ATOX1 delivers copper to ATP7A and ATP7B in the TGN [31]. Additionally, ATOX1 has been shown to function as a new type of copper-dependent transcription factor that mediates copper-induced cell proliferation [32].

CCS is a soluble cytoplasmic copper-chaperone protein that transfers copper ions to the copper-binding site of superoxide dismutase 1 (SOD1) [33]. SOD1 is a major antioxidant enzyme that catalyzes the conversion of superoxide radicals into oxygen and hydrogen peroxide, thereby maintaining reactive oxygen species (ROS) homeostasis and protecting the cells from oxidative stress damage [34]. This function has been shown in SOD1-knockout mice, where the absence of SOD1 increased oxidative stress and led first to liver cell damage and eventually liver cancer [35].

COX17 is responsible for delivering copper ions to the assembly of cytochrome c oxidase (COX), a key component of the electron transport chain involved in cellular respiration [36]. Subsequently, COX11 and SCO1, which are also important components of the COX assembly, donate copper to Cu(B) and Cu(A) sites in COX2 and COX1 core subunits of COX in the mitochondrial inner membrane, respectively. Additionally, COX17 also acts as a copper donor within the mitochondrial intermembrane space (IMS) [37]. COX17 is thus essential for proper COX assembly, with mutations in COX17 shown to further reduce COX activity, resulting in mitochondrial dysfunction and oxidative stress [38].

#### Intracellular copper storage and export

Intracellular copper export is mainly mediated by ATP7A and ATP7B, which are copper transporters located in the TGN that regulate copper delivery to secretory pathways and the plasma membrane [39]. Under normal conditions, ATP7A and ATP7B transport copper ions from the TGN to other cellular compartments for various cellular functions. At excessive intracellular copper levels, ATP7A and ATP7B are activated to export excess copper ions from the TGN and sequester them in copper-binding proteins such as metallothionein [40]. These proteins also regulate copper homeostasis by reducing uptake and increasing efflux. Mutations in ATP7A and ATP7B can lead to copper metabolism disorders such as Menkes disease and Wilson’s disease [41, 42].

## Evidence linking copper dyshomeostasis to atherosclerosis

Atherosclerosis is a chronic inflammatory disease characterized by plaque accumulation on the arterial walls, leading to narrowing and hardening of the arteries [43]. This process can result in serious complications, including coronary artery disease (CAD), stroke, and peripheral artery disease [44–46]. Growing evidence from numerous studies has linked copper dyshomeostasis and the resultant excess or deficiency of copper to atherosclerosis.

### Evidence linking copper excess and atherosclerosis

Extensive research has revealed a correlation between elevated copper levels and cardiovascular disease (Table 1). For instance, several prospective cohort studies have shown a significant correlation between elevated serum copper levels and higher mortality rates related to cardiovascular diseases, particularly coronary heart disease [47–49]. Elevated copper levels have also been shown to be an independent risk factor for ischemic heart disease [50]. In addition to evidence from prospective studies, Stadler et al. directly detected and quantified transition metal ions in human atherosclerotic plaques and found increased copper levels in the diseased intima [51]. Studies on populations with acute myocardial infarction (AMI) also support these findings, with patients with AMI exhibiting significantly higher serum copper levels than those without AMI [52]. Moreover, an increase in serum copper levels among post-MI patients was found to have considerable diagnostic value for the occurrence of MI [53]. Furthermore, altered copper bioavailability is negatively correlated with carotid intima-media thickness (IMT), which may serve as a reliable predictor for early atherosclerosis in patients with obesity [54]. Furthermore, serum copper concentrations differ among patients with different carotid atherosclerotic plaque morphologies. Specifically, patients with hemorrhagic plaques have significantly higher serum copper concentrations than those with calcified plaques [55]. These findings suggest a potential involvement of elevated copper levels in the pathogenesis of atherosclerosis.

**Table 1:** Evidence linking copper excess and atherosclerosis.

Country, author, year	Methods and study population	Result
Finland, Reunanen et al. [47]	230 men who died from CVDs and 298 matched controls; 10 years follow up.	The adjusted relative risk of CHD mortality between the highest and lowest tertiles of serum copper were 2.86 (*P* = 0.03). Elevated serum copper levels were significantly associated with an increased risk of mortality from all CVDs, especially from CHD.
United States, Ford, et al. [48]	4,574 participants aged ≥ 30 years; 151 died from CHD during 16-year follow-up.	There was an ~5% increase in serum copper levels among individuals who died from CHD compared to those who did not (*p* = 0.072). Elevated serum copper concentrations were associated with an increased risk of mortality from CHD.
Dutch, Kok et al. [49]	Cancer (*n* = 64) and CVD (*n* = 62) deaths, together with their respective matched controls, follow-up of six to 9 years.	Individuals with elevated serum copper levels (above 1.43 mg/liter) exhibited a roughly four times higher adjusted risk of mortality from cancer and CVD compared to those with serum copper levels within the normal range.
Eastern Finland, Salonen et al. [50]	1666 randomly selected men aged 42, 48, 54, or 60 years with no symptomatic IHD.	Elevated serum copper level is a risk factor for IHD that acts independently, as shown by a 3.5-fold to 4.0-fold increased risk of AMI associated with high serum copper levels (1.02-1.16 mg/liter and 1.17 mg/liter or more).
Nadina, Robyn et al. [51]	Atherosclerotic patients aged 67.9 ± 8.2 years and healthy controls.	Copper levels in the intima of lesions were notably higher in atherosclerotic patients compared to those in healthy controls (7.51 pmol/mg vs 2.01 pmol/mg tissue, *P* < 0.05).
Bangladesh, Begum et al. [52]	60 patients diagnosed with AMI and 60 healthy controls.	The mean serum copper level was significantly higher in AMI patients compared to that in controls (146.49 ± 23.52 μg/dl vs 105.44 ± 24.15 μg/dl, *p* < 0.05).
Grzegorz, Barbara et al. [53]	74 participants of MI and 72 healthy controls.	Higher serum copper level was significantly associated with MI (*P* < 0.001).
Italy, Tarantino et al. [54]	100 obese patients who had a low prevalence of comorbidities.	Altered copper bioavailability was negatively associated with carotid IMT (*t* = −2.23, *P* = 0.028) and may predict early atherosclerosis in obese patients.
Serbia, Tasić et al. [55]	91 patients (mean age 64 ± 7) with carotid atherosclerosis and 27 patients (mean age 58 ± 9) without carotid atherosclerosis.	Patients with hemorrhagic plaque had significantly higher serum copper levels compared to those with calcified plaque; high copper concentrations may contribute to atherosclerosis.

CVDs, cardiovascular disease; CHD, coronary heart disease; AMI, acute myocardial infarction; IHD, ischemic heart disease; IMT, intima-media thickness.

### Evidence linking copper deficiency and atherosclerosis

Copper deficiency is also recognized as a major contributing factor to the development of atherosclerosis. This is evidenced by the benefit of high dietary copper and copper supplementation. The Institute of Medicine recommends a daily dietary allowance of 900 ug of copper for adults, with a tolerable upper limit of 10,000 μg/day to prevent liver damage [56]. Although recommendations vary between national authorities, most recommend an intake of 800 to 2400 ug/day [15]. Substantial research suggests that adequate copper intake reduces the risk of atherosclerosis. For example, Rock et al. found that copper supplementation in middle-aged individuals enhanced the antioxidative capacity of cells, which may help prevent vascular damage and thus reduce the risk of atherosclerosis [57]. Another cohort study showed an association between adequate dietary intake of copper (equal to or above the estimated average requirement) and a reduced risk of all-cause- and cardiovascular disease-related mortality. However, this association was limited to copper intake from food sources [58]. Studies on animal models corroborate these findings with Lamb et al., showing that dietary copper deficiency or excess increases susceptibility to atherosclerosis of the aorta in cholesterol-fed rabbits [59]. Similarly, copper supplementation was found to reverse pathological changes induced by dietary iron overload in mice, partially normalizing cardiac hypertrophy [60] and improving cardiac function in pressure overload-induced dilated cardiomyopathy [61]. Nevertheless, the effects of copper supplementation on the cardiovascular system remain controversial. A study by Diaf et al. conducted on middle-aged women from Algeria showed that there is little association between dietary copper intake and atherosclerosis prevalence in diabetes [62]. These conflicting results may be due to differences in study design and the dose and duration of copper supplementation.

## Potential mechanisms of atherosclerosis development due to copper dyshomeostasis

The mechanisms of atherosclerosis development due to altered copper levels are not fully understood. Several potential mechanisms include oxidative stress, inflammation, endothelial dysfunction, and lipid metabolism.

### Oxidative stress

**Fig. 3:**
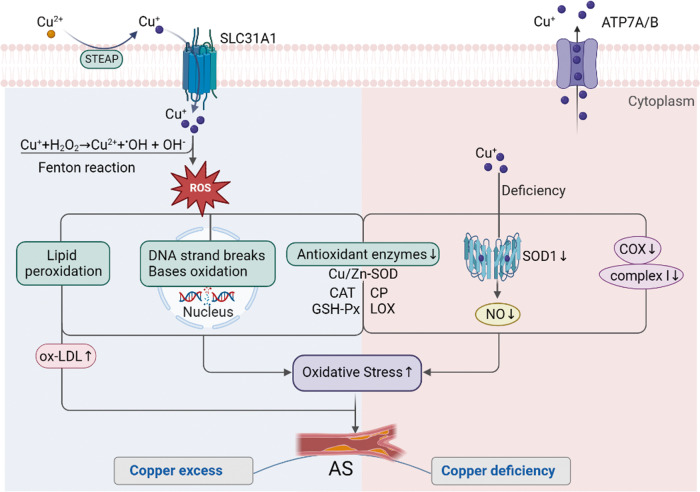
Copper dyshomeostasis promotes atherosclerosis through oxidative stress. Excessive copper interacts with H_2_O_2_ via the Fenton reaction, generating highly reactive hydroxyl radicals. These radicals induce lipid peroxidation, DNA strand breaks, and base oxidation and impair the function of antioxidant enzymes, ultimately leading to increased oxidative stress and potential damage to cells. On the other hand, copper deficiency also impairs the function of certain antioxidant enzymes, causing a decrease in SOD1 and COX activity. This reduced activity results in lowered NO levels, inactivation of complex I, and increased production of ROS, thereby exacerbating oxidative stress within cells. Together, these processes promote the development of atherosclerosis. Figure created with BioRender. SLC31A1 solute carrier family 31 member 1, STEAP six-transmembrane epithelial antigen of the prostate, H_2_O_2_ hydrogen peroxide, ^•^OH reactive hydroxyl radicals, ROS reactive oxygen species, ATP7A and ATP7B ATPase copper transporter 7A and 7B, CAT catalase, GSH-Px glutathione peroxidase, CP ceruloplasmin, LOX lysyl oxidase, SOD1 superoxide dismutase 1, COX cytochrome c oxidase, and AS atherosclerosis.

Oxidative stress is a key factor in atherosclerosis development, which typically involves an imbalance in reactive oxygen species (ROS) production and antioxidant defenses [63]. Since copper is a redox-active metal, changes in copper levels can contribute to the generation of ROS and thereby promote oxidative stress [15, 64] **(**Fig. 3**)**.

#### Copper excess and oxidative stress

Elevated levels of free copper ions tend to interact more with hydrogen peroxide through Fenton reactions, leading to the production of highly-reactive hydroxyl radicals [65]. These radicals cause lipid peroxidation, protein oxidation, and DNA damage, ultimately contributing to the initiation and progression of atherosclerosis [66]. Lipid peroxidation is a chain reaction initiated by an attack of ROS on polyunsaturated fatty acids in cell membrane lipids, which then leads to the oxidative damage of lipid molecules. Increased copper levels can promote lipid peroxidation and result in the formation of oxidized low-density lipoprotein (ox-LDL), which is an atherosclerosis risk factor [67]. Furthermore, increased copper levels have been shown to reduce the activity of antioxidant enzymes, such as Cu/Zn-SOD, catalase, and glutathione peroxidase, in the red blood cells, whole blood, live tissue, and brain tissue of rats (67, 68, 69). In rat brain tissue, the decrease in SOD activity and glutathione levels arises because copper overload induces lipid peroxidation [68]. Copper is also capable of causing DNA strand breaks and the oxidation of bases via oxygen-derived free radicals [69]. These findings suggest that copper overload causes impairment of the antioxidant defense system in several tissues.

#### Copper deficiency and oxidative stress

Copper deficiency has also been suggested to contribute to the development and progression of atherosclerosis via oxidative stress. Copper is an essential cofactor of various antioxidant enzymes, including Cu/Zn-SOD, ceruloplasmin, and lysyl oxidase [70]. Copper deficiency may thus impair the function of these enzymes, leading to an impaired antioxidant defense system and increased susceptibility to oxidative stress [70, 71]. This suggestion is supported by copper deficiency in rats being found to reduce Cu/Zn-SOD activity and increase oxidative damage to various subunits of the erythrocyte spectrin [72]. Decreased activity of *SOD1*, which encodes Cu/Zn SOD, has also been observed in the liver and red blood cells of copper-deficient rats [73]. The decrease in *SOD1* caused by copper deficiency leads to a reduction in NO levels, which may promote endothelial dysfunction, reduce vascular relaxation, and increase oxidative stress, thus ultimately contributing to atherosclerosis development [70]. In addition to affecting antioxidant enzymes, copper deficiency may also reduce COX activity and lead to oxidative inactivation of complex I (NADH: ubiquinone oxidoreductase). This oxidative inactivation may then lead to elevated production of ROS in copper-deficient cells, thereby exacerbating cellular oxidative stress [74].

### Inflammation

Copper may also promote atherosclerosis development by modulating inflammatory responses associated with the disease.

#### Copper excess and inflammation

Excess copper has been implicated in atherosclerosis pathogenesis due to its ability to induce inflammation. High copper levels were previously reported to stimulate pro-inflammatory cytokine production and thereby promote inflammation within arterial walls [75]. For example, in primary cardiac cells, Cu^2+^ increases the release of interleukin-6 (IL-6) and activates MAP kinases, which are linked to cardiac inflammation and hypertrophy [76]. Copper-induced oxidative stress also contributes to inflammation because excessive copper generates ROS, which causes oxidative damage to lipids, proteins, and DNA and promotes inflammation [15]. Furthermore, increases in ROS levels due to increased copper levels can, in turn, lead to the activation of nuclear factor-κB (NF-κB), a crucial protein involved in regulating the expression of proinflammatory genes [77]. These findings suggest that high copper levels can exacerbate inflammation of the vascular walls and thus promote atherosclerosis development.

#### Copper deficiency and inflammation

Copper deficiency has also been linked to atherosclerosis development through its impact on inflammation. Copper deficiency may result in decreased expression of adhesion molecules, such as ICAM-1 and VCAM-1, which facilitate leukocyte adhesion onto activated endothelial cells [78]. Evidence from animal studies also supports the role of copper deficiency in inflammation. For example, neutrophil accumulation increases due to increased ICAM-1 expression in the livers of copper-deficient rats following ischemia/reperfusion [79]. Further, this elevated ICAM-1 expression has been shown to activate neutrophils and endothelial cells, as evidenced by F-actin polymerization and increased accumulation of neutrophils within the lung microcirculation [80–82]. Pulmonary inflammatory responses are also intensified in copper-deficient animals [83]. Finally, copper deficiency impairs the activity of Cu/Zn-SOD as previously mentioned, leading to an increased accumulation of ROS and exacerbation of oxidative stress, which can further promote inflammation and atherosclerosis development [84].

### Endothelial dysfunction

Copper dyshomeostasis has been linked to atherosclerosis, with both an excess and deficiency of copper contributing to endothelial dysfunction, a critical early step in atherogenesis.

#### Copper excess and endothelial dysfunction

Excessive copper can disrupt the balance between the production and degradation of nitric oxide (NO), a key regulator of vascular tone and endothelial function [85]. Increased copper levels can upregulate inducible NO synthase (iNOS) expression, resulting in excessive NO production and peroxynitrite, a potent oxidant that causes further oxidative damage [86]. Moreover, copper also interacts with atherosclerosis risk factors such as homocysteine and thereby causes increased hydrogen peroxidation and oxidative stress. Specifically, a study shows that incubation with homocysteine and copper for 4 h is able to cause significant damage to endothelial cells [87].

#### Copper deficiency and endothelial dysfunction

Copper deficiency is associated with increased endothelial dysfunction as well. Endothelial dysfunction can result in decreased production of NO, a vasodilator and inhibitor of platelet aggregation, and thereby promote a proatherogenic environment [88]. Copper deficiency can also lead to decreased NO levels by reducing the levels of SOD1. Reduced NO levels can then inhibit endothelial function and vasodilation, increase oxidative stress, and thereby promote atherosclerosis [70].

### Lipid metabolism

#### Copper excess and lipid metabolism

Excess copper strongly contributes to atherosclerosis development via its effects on the ox-LDL, which plays a central role in atherosclerosis [89]. Specifically, copper participates in the oxidation of LDL particles, and alterations in copper levels may affect the susceptibility of LDL particles to oxidation. Excess copper levels can increase the oxidation of LDL particles and trigger the production of ox-LDL, which then contributes to atherosclerosis development [67].

In addition to its effects on ox-LDL, copper is involved in several forms of lipid metabolism, including fatty acid and cholesterol synthesis and lipoprotein metabolism. Alterations in copper levels may be associated with changes in lipid and lipoprotein concentrations. For example, serum copper and ceruloplasmin levels were found to be positively associated with lipid peroxides, total cholesterol, triglycerides, and apolipoprotein B in healthy individuals [90]. In another study, the serum copper levels of Iranian patients with angiographically defined CAD were found to be positively correlated with fasting serum triglycerides [91]. In vivo, evidence from animal experiments also supports the role of excess copper in lipid metabolism. A study on yellow catfish demonstrated that copper-induced endoplasmic reticulum stress and disrupted calcium homeostasis alter hepatic lipid metabolism, leading to increased lipid accumulation in the liver [92].

#### Copper deficiency and lipid metabolism

Insufficient copper levels may also contribute to atherosclerosis development by affecting lipid metabolism. Since copper is involved in the synthesis of fatty acids and cholesterol, insufficient copper levels may lead to impaired lipid metabolism and, thus, the development of fatty liver disease. Copper deficiency increases total cholesterol levels as well [7]. Insufficient copper levels can affect the activity of sterol regulatory element-binding proteins 1 and 2 (SREBP-1 and SREBP-2), which are transcription factors involved in fatty acid and cholesterol metabolism [93]. Specifically, both SREBP-1 isoforms (SREBP-1a and SREBP-1c) are involved in the regulation of fatty acid synthesis, whereas SREBP-2 is mainly involved in the regulation of cholesterol biosynthesis [94]. Low copper diets were found to induce accumulation of the mature form of SREBP-1 in the nuclei of rat liver cells, yet no change was observed in the DNA-binding site of SREBP-1 [95]. Hence, copper may play a role in regulating the subcellular localization of SREBP-1, potentially affecting its activity and, in turn, lipid metabolism. Further research is needed to fully understand the mechanisms through which copper affects the activity of SREBP-1 and other transcription factors involved in lipid metabolism.

### Other risk factors

Copper levels are also associated with other atherosclerosis risk factors, such as high blood pressure. Copper inhibits the activity of angiotensin-converting enzymes, such that copper deficiency leads to increasing angiotensis levels and, thus, water and sodium retention and hypertension [96]. Moreover, copper is a cofactor of SOD, which is a major player in the antioxidant defense system. Hence, copper deficiency may increase the levels of superoxide free radicals, leading to elevated angiotensin levels and consequent hypertension [97].

## Copper-induced cell death and atherosclerosis

### From copper-induced cell death to cuproptosis

The discovery of copper-induced cell death dates back to the early 1980s [98]. Increased copper levels were found to promote ROS generation and thereby lead to oxidative stress, DNA damage, and, ultimately cell death [99]. These findings have led to further investigations into the molecular mechanisms underlying copper-induced cell death and their potential implications for human diseases. Indeed, conflicting findings suggest that, in addition to ROS accumulation, excess copper may induce cell death through apoptosis or caspase-independent cell death [100, 101]. Overall, the mechanisms responsible for copper-induced cell death were not well understood.

**Fig. 4:**
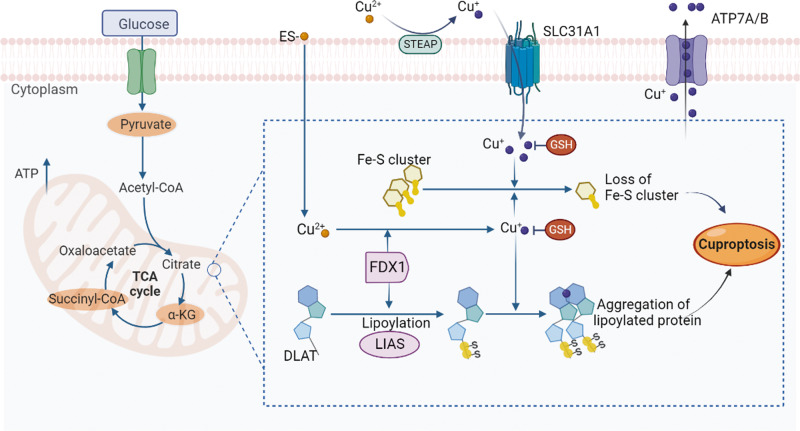
Schematic diagram of the mechanisms of cuproptosis. The copper ionophore ES transports extracellular copper into the cell. Subsequently, intracellular copper binds to lipoylated mitochondrial enzymes (such as DLAT) involved in the TCA cycle. This binding leads to the aggregation of these proteins, which can disrupt the TCA cycle and, therefore, interfere with cellular energy production. The upstream regulatory factors FDX1 and LIAS play a critical role in this process. The aggregation of lipoylated mitochondrial proteins and loss of Fe-S clusters promote proteotoxic stress and ultimately lead to cell death. Figure created with BioRender. SLC31A1 solute carrier family 31 member 1, STEAP the six-transmembrane epithelial antigen of the prostate, ATP7A and ATP7B ATPase copper transporter 7A and 7B, ES elesclomol, α-KG α-ketoglutarate, DLAT dihydrolipoamide S-acetyltransferase, FDX1 ferredoxin-1, Fe–S iron–sulfur, LIAS lipoic acid synthetase, TCA tricarboxylic acid, and GSH glutathione.

However, in March 2022, Tsvetkov et al. published a groundbreaking study unveiling the mechanism of copper-induced cell death, which was termed cuproptosis [12]. Cuproptosis represents a unique form of cell death triggered by an excess of copper ions. In their study, Tsvetkov et al. showed that treatment with the copper ionophore elesclomol induced cell death. Remarkably, only the copper chelator can rescue cells from elesclomol-induced cell death. In contrast, rescue is not possible with any of the inhibitors for apoptosis, necroptosis, oxidative stress, ROS induced cell death, or ferroptosis. These findings unequivocally establish that cuproptosis is distinct from other known cell death modalities, underscoring its unique mechanisms and signaling pathways. Notably, cuproptosis is regulated by mitochondrial respiration, as supported by research indicating that mitochondria-dependent cells exhibit a sensitivity to copper ionophores nearly 1,000 times higher than cells undergoing glycolysis [12]. The importance of mitochondrial respiration in cuproptosis has been highlighted in further research, revealing a close correlation between cuproptosis and the tricarboxylic acid (TCA) cycle. During cuproptosis, intracellular copper binds to the lipoylated components of the TCA cycle. This leads to the aggregation of copper-bound lipoylated mitochondrial proteins, which can disrupt the TCA cycle and, therefore, interfere with cellular energy production. The upstream regulatory factors FDX1 and LIAS are critical in this process. Aggregation of these proteins and the subsequent reduction of Fe–S clusters, which are essential cofactors for various cellular processes, including electron transport and enzymatic reactions [102], promote proteotoxic stress and ultimately lead to cell death **(**Fig. 4**)**.

### Potential interactions between copper-induced cell death and other cell death pathways

Accumulating evidence suggests widespread cross-talk and interactions among the primary initiators, effectors, and executioners involved in pyroptosis, necroptosis, ferroptosis, and cuproptosis.

#### Pyroptosis

Pyroptosis is a pro-inflammatory programmed cell death that is primarily driven by inflammasome assembly, accompanied by GSDMD cleavage and IL-1β and IL-18 release [103–105]. NLRP1, NLRP3, NLRC4, AIM2, and pyrin are well-established inflammasome sensors that assemble canonical inflammasomes in a process induced by inflammatory stimuli such as those resulting from microbial infections [106, 107]. Copper ions, which are essential micronutrients for many physiological processes, have been shown to trigger ROS production and activate the NF-κB signaling pathway. This leads to the upregulation of pro-inflammatory genes and cytokines, potentially influencing the progression of atherosclerosis [108–110]. Therefore, there is a plausible suggestion of crosstalk between copper ions and pyroptosis.

Evidence from animal studies demonstrates that copper loading in rat hepatocytes leads to a significant increase in the mRNA expression of pyroptosis-related genes (caspase-1, IL-18, IL-1β, and NLRP3) and the protein expression of caspase-1[111]. Similarly, copper exposure in mouse microglial cells triggers an inflammatory response, resulting in the upregulation of NLRP3/caspase 1/GSDMD axis proteins and subsequent pyroptosis. These effects are likely mediated by the early activation of the ROS/NF-κB pathway and subsequent disruption of mitophagy [77]. Moreover, comparable outcomes were observed in murine macrophages treated with copper oxide nanoparticles. Copper oxide nanoparticle exposure induces oxidative stress and activates NLRP3 inflammasomes, leading to the expression of pro-IL-1β through the MyD88-dependent TLR4 signaling pathway, followed by NF-κB activation in murine macrophages [108].

In summary, these findings demonstrate the presence of crosstalk between copper-induced cell death and pyroptosis. Copper exposure influences gene and protein expression associated with pyroptosis in various cell types, with the underlying mechanisms being ROS/NF-κB pathway activation and the inflammatory response. Further research is needed to elucidate the precise underlying mechanisms and explore the implications of this interaction.

#### Necroptosis

Necroptosis is a form of programmed necrosis linked to atherosclerosis via its potential involvement in plaque destabilization and rupture [112, 113]. Necroptosis was recently shown to activate the NLRP3 inflammasome by causing potassium efflux through MLKL pores in macrophages [114]. Bioinformatics analysis further indicated an association between ZBP1 and cuproptosis as well as necroptosis [115]. ZBP1 activation led to the recruitment of RIPK3 and caspase-8 to activate the NLRP3 inflammasome, which in turn triggers necroptosis and pyroptosis [116, 117].

#### Ferroptosis

Ferroptosis, a form of iron-dependent cell death triggered by lipid peroxidation and accumulation of lipid-based reactive oxygen species [118–120], appears to be influenced by copper levels due to the redox-active properties of copper ions [121, 122]. Cuproptosis can modulate the expression of key genes involved in ferroptosis regulation, such as glutathione peroxidase 4 (GPX4), by eliminating phospholipid hydroperoxides [123, 124] and acylcoenzyme A synthetase long-chain family member 4 (ACSL4) [125], thereby regulating the sensitivity of cells to ferroptosis inducers. Notably, copper chelators can reduce sensitivity to ferroptosis specifically while leaving other forms of cell death unaffected. In a study by Xue et al., copper was found to play a novel role in promoting ferroptotisis through the degradation of GPX4 via macroautophagy/autophagy [126]. Furthermore, research by Gao et al. reveals an interaction between copper-induced cell death and necroptosis. Their findings indicate that elesclomol administration to colorectal cancer cells increased Cu(II) levels in the mitochondria, downregulated ATP7A expression, and increased ROS accumulation. This process triggered SLC7A11 degradation, intensifying oxidative stress and resulting in ferroptosis [127]. Additionally, copper depletion greatly enhanced ferroptosis through mitochondrial perturbation and the disruption of antioxidant mechanisms [128]. Specifically, copper depletion limits GPX4 protein expression and reduces cellular sensitivity to ferroptosis inducers, establishing a direct link between copper levels and ferroptosis [128].

Based on the aforementioned studies, it is evident that copper is closely linked to necroptosis, pyroptosis, and ferroptosis, suggesting a significant cross-talk between these different cell death pathways. Understanding the underlying mechanisms that connect these modes of cell death is of paramount importance for the development of novel atherosclerotic therapeutic strategies that can target multiple pathways simultaneously.

### Cuproptosis-related mitochondrial dysfunction and atherosclerosis

Mitochondria are vulnerable to copper-induced damage, which causes oxidative damage to its membrane [129, 130]. Excessive intracellular copper may also disrupt mitochondrial function by altering the activity of several key enzymes, such as those involved in the tricarboxylic acid (TCA) cycle and oxidative phosphorylation [131]. Severe mitochondrial dysfunction and decreases in the activities of several liver enzymes, including complex I, complex II, complex III, complex IV, and aconitase, were observed in patients with copper overload. These effects are potentially mediated by the accumulation of copper in the mitochondria [132]. Furthermore, copper treatment induces selective changes in metabolic enzymes through lipoylation, which is a highly conserved lysine post-translational modification. Protein lipoylation occurs only on Dihydrolipoamide S-Acetyltransferase (DLAT), Dihydrolipoamide S-Succinyltransferase (DLST), Dihydrolipoamide Branched Chain Transacylase E2 (DBT), and Glycine Cleavage System Protein H (GCSH), all of which are involved in metabolic complexes that regulate carbon entry points to the TCA cycle [133, 134]. Additionally, copper overload may trigger the opening of the mitochondrial permeability transition pore and cause the release of pro-apoptotic factors, ultimately resulting in cell death [135].

Mitochondrial dysfunction is implicated in atherosclerosis development and progression [136]. Impaired mitochondrial function promotes lipid accumulation, oxidative stress, inflammatory responses, and proliferation of vascular smooth muscle cells, all of which contribute to plaque formation and destabilization [137, 138]. Considering its impact on mitochondrial function, cuproptosis may contribute to the progression of atherosclerosis by aggravating mitochondrial dysfunction.

## Potential therapeutic strategies targeting cuproptosis in atherosclerosis

### Copper chelators

Copper chelators bind and sequester copper ions. Several copper chelators have shown promise in animal studies and clinical trials for atherosclerosis treatment.

Tetrathiomolybdate (TTM) has been shown to be an effective copper chelator with potential therapeutic implications in attenuating atherosclerosis progression, as demonstrated in animal models [139, 140]. In a study using ApoE^-/-^ mice, TTM treatment for 10 weeks significantly reduced aortic lesion development, indicating its potential as an anti-atherosclerotic agent [139]. The beneficial effects of TTM were attributed to its ability to reduce bioavailable copper levels and inhibit vascular inflammation [140]. Copper is involved in vascular inflammation as an etiologic factor of atherosclerotic vascular disease, and by chelating copper, TTM may modulate copper-related pathways involved in atherosclerosis and inflammation. Wei et al. demonstrated that TTM copper chelation inhibited lipopolysaccharide (LPS)-induced inflammatory responses in mice aorta and other tissues. This inhibition may occur by suppressing redox-sensitive transcription factors NF-κB and AP-1, which play crucial roles in inflammation [140]. By targeting copper-related pathways and modulating inflammatory processes, TTM exhibits potential as an anti-atherosclerotic agent.

Ethylenediaminetetraacetic acid disodium salt (EDTA) is a broad-spectrum metal-chelating agent that has also been investigated for its potential to treat atherosclerosis [141]. For instance, a double-blind placebo-controlled trial (*N* = 1708) shows that chelation therapy with disodium EDTA reduced the risk of adverse cardiovascular outcomes in stable patients with a history of myocardial infarction (MI). The primary endpoint occurred less frequently in the chelation group than in the placebo group (26% vs. 30%) [142]. These findings offer preliminary evidence to direct further studies but do not in themselves provide sufficient evidence to justify the routine use of chelation therapy in patients with MI. Another meta-analysis of five studies covering a total of 1,993 randomized participants failed to identify sufficient evidence to determine the effectiveness of chelation therapy in the treatment of atherosclerotic cardiovascular disease [143]. The contradictory results among these studies may be attributed to variations in research design and/or characteristics of the study populations. Therefore, further research is needed before routine use of chelation therapy can be recommended.

### Regulation of copper chaperone protein expression

Copper chaperones play a crucial role in maintaining cellular copper homeostasis by facilitating the transfer of copper ions to target proteins and organelles. Modulating the expression of copper chaperone proteins potentially offers an alternative approach to regulate copper levels and limit the contribution of copper-induced cell death to atherosclerosis.

ATOX1 is a copper chaperone protein that is of major importance in mammalian cells. ATOX1 has been shown to translocate to the nucleus in response to inflammatory cytokines or exogenous copper. Furthermore, ATOX1 is localized in the nucleus of endothelial cells in the inflamed atherosclerotic aorta [144]. The migration of VSMCs is crucial for neointimal formation following vascular injury and atherosclerotic lesion formation. ATOX1 was found to promote VSMC migration and inflammatory cell recruitment to injured vessels [145]. Furthermore, copper-dependent binding of ATOX1 to TRAF4 is required to facilitate nuclear translocation of ATOX1 and ROS-dependent inflammatory responses in TNF-α-stimulated endothelial cells (ECs) [146]. This highlights the potential of targeting the ATOX1-TRAF4 axis as a novel therapeutic strategy for the treatment of atherosclerosis. In summary, ATOX1 represents a promising therapeutic target for inflammation-related vascular diseases such as atherosclerosis.

### Copper ionophores

Copper ionophores transport copper into the cells, leading to an increase in intracellular copper levels and subsequent cell death. However, copper chelators can inhibit this process. Several drugs can act as copper ionophores, including disulfiram, pyrithione, chloroquine, and elesclomol [147]. Among these, elesclomol has received the most attention and has been subjected to several clinical trials for use in cancer treatment. Although the majority of these trials have not shown promising results regarding further development of elesclomol as a drug, they have verified its safety [148]. Nanomedicines combining copper ions with copper ionophores are also currently being widely investigated. Recently, the researchers successfully designed multifunctional nanoparticles with pH-responsive and CD44-targeted properties. The utilization of dendritic mesoporous silica nanoparticles capped with copper sulfide and coated with hyaluronic acid enables precise drug delivery and controlled release in the acidic microenvironment of atherosclerotic inflammation [149]. These findings highlight the potential of copper-based nanomedicines in developing innovative approaches for targeted atherosclerosis therapy. Notably, an important aspect to consider in the advancement of copper ionophores for clinical therapeutic applications is the notable impact that slight structural changes can exert on their properties and functions [16]. In addition, copper ionophore-mediated cell death is strongly correlated with mitochondrial metabolism and closely associated with atherosclerosis development. Based on these findings, copper ionophores may represent a novel therapeutic strategy for targeting copper-induced cell death in atherosclerosis.

## Conclusions and future perspectives

In conclusion, the multifaceted role of copper in atherosclerosis involves complex cellular and systemic metabolic processes. Copper deficiency or excess promotes atherosclerosis by inducing oxidative stress, inflammation, endothelial dysfunction, and adverse effects on lipid metabolism. Further research is required to elucidate the interactions between these factors and their role in the development of atherosclerotic lesions.

Copper-induced cell death and the subsequent emergence of the concept of cuproptosis have expanded our understanding of the role of copper in atherosclerosis. The involvement of cuproptosis and the associated mitochondrial dysfunction in atherosclerosis highlights the need for further research into the molecular mechanisms underlying these processes. Therapeutic targets and strategies for mitigating the detrimental effects of copper-induced cell death in atherosclerosis have also been identified, including modulating copper levels, inhibiting cuproptosis, or enhancing cellular defenses against cuproptosis. However, potential side effects should also be considered since an excessive reduction in copper levels may impair essential physiological processes or, in itself, lead to atherosclerosis. Additionally, the absence of cuproptosis-specific biomarkers is a significant barrier that hinders further development of clinical applications regarding cuproptosis. Identifying such biomarkers may facilitate risk stratification and the development of personalized therapeutic approaches.
